# Key facets to build up eHealth and mHealth interventions to enhance physical activity, sedentary behavior and nutrition in healthy subjects – an umbrella review

**DOI:** 10.1186/s12889-020-09700-7

**Published:** 2020-10-23

**Authors:** Janis Fiedler, Tobias Eckert, Kathrin Wunsch, Alexander Woll

**Affiliations:** Institute of Sports and Sports Science, Karlsruhe Institute of Technology, Engler-Bunte-Ring 15, 76131 Karlsruhe, Germany

**Keywords:** Telemedicine, Health behavior, Primary prevention, Exercise, Sedentary behavior, Food and nutrition, Umbrella review, Psychology social, Just-in-time adaptive intervention, Psychological theory

## Abstract

**Background:**

Electronic (eHealth) and mobile (mHealth) health interventions can provide a large coverage, and are promising tools to change health behavior (i.e. physical activity, sedentary behavior and healthy eating). However, the determinants of intervention effectiveness in primary prevention has not been explored yet. Therefore, the objectives of this umbrella review were to evaluate intervention effectiveness, to explore the impact of pre-defined determinants of effectiveness (i.e. theoretical foundations, behavior change techniques, social contexts or just-in-time adaptive interventions), and to provide recommendations for future research and practice in the field of primary prevention delivered via e/mHealth technology.

**Methods:**

PubMed, Scopus, Web of Science and the Cochrane Library were searched for systematic reviews and meta-analyses (reviews) published between January 1990 and May 2020. Reviews reporting on e/mHealth behavior change interventions in physical activity, sedentary behavior and/or healthy eating for healthy subjects (i.e. subjects without physical or physiological morbidities which would influence the realization of behaviors targeted by the respective interventions) were included if they also investigated respective theoretical foundations, behavior change techniques, social contexts or just-in-time adaptive interventions. Included studies were ranked concerning their methodological quality and qualitatively synthesized.

**Results:**

The systematic search revealed 11 systematic reviews and meta-analyses of moderate quality. The majority of original research studies within the reviews found e/mHealth interventions to be effective, but the results showed a high heterogeneity concerning assessment methods and outcomes, making them difficult to compare. Whereas theoretical foundation and behavior change techniques were suggested to be potential positive determinants of effective interventions, the impact of social context remains unclear. None of the reviews included just-in-time adaptive interventions.

**Conclusion:**

Findings of this umbrella review support the use of e/mHealth to enhance physical activity and healthy eating and reduce sedentary behavior. The general lack of precise reporting and comparison of confounding variables in reviews and original research studies as well as the limited number of reviews for each health behavior constrains the generalization and interpretation of results. Further research is needed on study-level to investigate effects of versatile determinants of e/mHealth efficiency, using a theoretical foundation and additionally explore the impact of social contexts and more sophisticated approaches like just-in-time adaptive interventions.

**Trial registration:**

The protocol for this umbrella review was a priori registered with PROSPERO: CRD42020147902.

**Supplementary information:**

**Supplementary information** accompanies this paper at 10.1186/s12889-020-09700-7.

## Background

Physical activity (PA), a reduction of sedentary behavior (SB) and healthy eating (i.e. enhanced fruit and vegetable intake (FVI), reduced sugar and saturated fat intake among others) (HE) are key strategies in the primary prevention of noncommunicable diseases like cardiovascular diseases, diabetes, cancer and obesity, which were responsible for 41 million deaths worldwide in 2016 [1]. Despite this knowledge, the levels of PA and HE are often insufficient in our modern society throughout all age groups [2–6], while SB, such as excessive sitting during worktime (e.g. deskwork) and during leisure time (e.g. watching television), increased over the past years [7, 8]. As a result, guidelines concerning PA, SB and HE are put into place, but the sole presence of these recommendations is not sufficient to change health behavior and to reduce the financial and health burden worldwide [9]. Working towards achieving these guidelines is important throughout all stages of life and can be seen as a long-term investment which seems to be easier to achieve for healthy people since obesity or other morbidities add further barriers which restrict engagement in healthy behaviors [10]. Focusing on primary prevention in healthy participants can therefore be a sustainable way to reduce the prevalence of noncommunicable diseases. One promising strategy for primary care prevention might be the usage of electronic (eHealth) and mobile (mHealth) health interventions. eHealth interventions comprise “the use of information and communication technologies for health” [11], while mHealth interventions refer to “medical and public health practice supported by mobile devices, such as mobile phones, patient monitoring devices, personal digital assistants, and other wireless devices” [12]. With 4.5 billion active internet users in 2020 worldwide [13], the potential coverage of e/mHealth tools coupled with intuitive and autonomous control of the device by the end user hold great promise. This is especially true for the younger and digital native generations who are known to interact frequently with e/mHealth [14]. For the establishment of e/mHealth in primary prevention, several methodological issues such as the need for accurate and validated measuring tools for a better comparison of different e/mHealth approaches and dose/response relationship for interventions require further investigation [15].

Theoretical foundation of interventions, as depicted by behavior change theories (e.g. self-determination theory [16], theory of planned behavior [17], transtheoretical model [18] or social cognitive theory [19]), and by behavior change techniques (BCTs) [20, 21] were shown to be important facets for intervention effectiveness [22, 23]. Additionally, health behaviors are usually linked to social contexts and affected by social relations [24]. Thus, facets like information about and interacting with other users or peers [25] might also have an important impact on intervention effectiveness and might help to sustain successful behavior change [26]. This has been especially true for adolescents as their sufficient level of PA, SB and HE strongly depend on their families, schools and peers [27]. Therefore, the integration and documentation of social contexts is important to assess the influence on and enhance the effectiveness of sustainable health behavior change. Furthermore, individual tailoring based on theoretical constructs was shown to be positively associated with effective interventions [25]. Delivering these interventions during the most promising time for the desired behavior (e.g. PA and HE) or during the most vulnerable time for unhealthy behavior (e.g. SB), implementation of the so called just-in-time (adaptive) intervention (JITAI) [28, 29] and ecological momentary intervention (EMI) [30] are promising new approaches for effective e/mHealth interventions. With the development of new generations of a variety of sensors [28] and the integration of machine learning approaches [31], the advances in individual tailoring are rapidly evolving and appear to be auspicious facets to implement in behavior change interventions.

Existing umbrella reviews concerning mHealth in general revealed only limited evidence to be effective to change a variety of behaviors [32], while the use of text messages has shown effectiveness for several health outcomes [33]. There is an abundance of mHealth interventions for diabetes which led to clinically relevant improvements [34, 35]. Existing umbrella reviews in the area of digital behavior change interventions expressed the need to examine the key contents of effective interventions in different settings (e.g. home, work or school based interventions) [36], and to consider various facets for an effective implementation [37]. An overview of efficient intervention components has only been composed for non-e/mHealth interventions promoting PA, SB and HE [38–41]. Key determinants of effectiveness in these overviews were the use of theoretical foundations [38, 40, 41], BCTs [40], social contexts [38–41] and using prompts and feedback [38, 40, 41]. Taken together, there is a research gap for e/mHealth interventions concerning facets of effectiveness with a focus on health behavior change in primary prevention.

In order to determine if these facets (i.e. theoretical foundations, BCTs, social contexts, JITAIs) were incorporated in recent e/mHealth interventions of primary prevention and with which magnitude they contributed to intervention effectiveness (in addition to methodological facets), a systematic summary of research by conducting an umbrella review [42] is needed.

## Methods

This umbrella review was registered a priori with PROSPERO (International prospective register of systematic reviews, registration number CRD42020147902). It was conducted based on the Preferred Reporting Items for Systematic Reviews and Meta-Analyses (PRISMA) statement [43].

### Study aim

The present umbrella review aimed to systematically summarize the results from systematic reviews and meta-analyses concerning the effectiveness of e/mHealth interventions to promote PA, reduce SB and promote HE as a primary care strategy in healthy participants. Further, the umbrella review aims to identify the impact of theoretical foundations, BCTs, social contexts and JITAIs on the effectiveness of e/mHealth interventions. Moreover, the recommendations for future research provided by the included reviews were analyzed and expanded to provide an overview of needs to be addressed in future developments of e/mHealth interventions.

### Data sources and search strategy

A systematic search for reviews published in English between 01.01.1990 and 16.08.2019 was conducted using the four databases PubMed, Scopus, Web of Science and the Cochrane Library for systematic reviews and meta-analyses. The search was conducted by one author and repeated prior to submission on 20.05.2020 (JF). The search terms were reviewed by two authors (JF, KW) and included the following key constructs as well as numerous synonyms thereof: (eHealth OR mHealth) AND (PA OR SB OR HE) AND (theoretical foundation/BCT OR social OR JITAI/EMI). Additionally, a forward- / backward-search was conducted on the reference lists of included reviews. Please see additional file 1 for detailed search strategy of all databases.

### Review selection

**Fig. 1:**
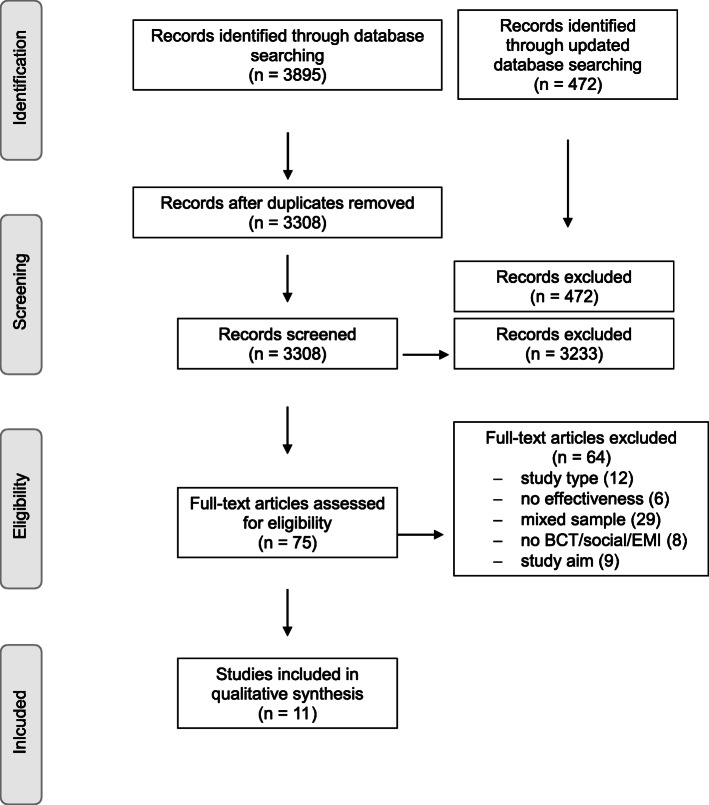
PRISMA Flow Chart of study selection process

Following the systematic search, literature was imported to the reference management software *CITAVI* 6. After duplicates were removed, two reviewers (JF, KW) independently examined titles and abstracts. Full texts of relevant review articles were obtained and assessed based on the inclusion and exclusion criteria described below (JF, TE). Reasons for exclusion at this stage were recorded and are displayed in the PRISMA-flow chart (Fig. 1). Any disagreements between authors were resolved by consensus and/or discussion with a third author (KW or TE).

#### Inclusion and exclusion criteria

Inclusion and exclusion criteria were selected based on PICOS (1. Population, 2. Intervention, 3. Comparison, 4. Outcome and 5. Study type) [43].

1. Population inclusion: Healthy participants of all ages with no physical or physiological morbidities including obesity (BMI > 30 kg/m^2^) which would influence the realization of behaviors (i.e. PA, SB and HE) targeted by the respective interventions. If a review included patient groups or participants with any physical or psychological morbidities and provided a subgroup analysis or reported the results for the healthy population separately, the review was also included. Exclusion: Participants with any physical or psychological morbidities including obesity (BMI > 30 kg/m^2^), clinical settings and studies focusing on populations, whose PA, SB or HE was influenced by disease specific recommendations or health status.
2. Intervention inclusion: e/mHealth interventions where the primary outcome measure was PA (e.g. steps, moderate, vigorous or moderate to vigorous (MVPA)) and/or SB (e.g. sitting time, screen time) and/or HE (e.g. FVI, fat consumption) were selected. Exclusion: Studies without an intervention, with no e/mHealth interventions, with mixed interventions if e/mHealth was not analyzed separately.
3. Comparison: Included reviews were not limited to comparator studies.
4. Outcome inclusion: Effectiveness for PA and/or SB and/or HE as the main outcome. Effectiveness had to be displayed or discussed with regard to at least one of the following aspects: a) theoretical foundation or BCTs, b) JITAI/EMI, c) social context (e.g. social network, family/peer group/school setting). Exclusion: Studies that focused on other health outcomes like weight loss, quality of life, or had multiple additional health behaviors not related to PA, SB or HE (e.g. smoking, drinking) as main outcomes. Studies without discussion/results for any of the following: theoretical foundation or BCTs, JITAI/EMI, or social context.
5. Study type inclusion: Systematic reviews and meta-analyses based on the PRISMA statement. Exclusion: Non-systematic reviews (e.g. narrative reviews, qualitative reviews, scoping reviews).

### Study quality assessment

Review quality was rated independently by one author (JF) and a research assistant using the assessment of multiple systematic reviews (AMSTAR) tool [44]. Any disagreements were resolved by discussion until a common consent was found.

### Data extraction

Data was extracted from the included studies by JF using a predefined Excel sheet. Data extraction and coding were checked by a research assistant. Any disagreements were resolved by discussion until a common consent was found. The following data were extracted: author and year, type of review, aim, mHealth/eHealth tools, country (where the included studies were conducted), main outcomes (constructs and parameters), time period searched and time period of included studies, included study designs, number of studies, number and age of participants, intervention duration, quality of included studies indicated by the reviews, included theory/BCT, included social/JITAI/EMI, reported effectiveness, recommendations for future research as stated by the authors.

### Analysis

We used the term review to describe systematic reviews and meta-analysis together, and distinguished between the terms study and publication, since the reviews included multiple publications about one study and thus relate to the same sample. Due to the heterogeneity of methods and reported values, a quantitative pooling of data was not feasible. Descriptive data were extracted and displayed as rounded percentage for a better comparison (e.g. 12/20 (55%) studies were effective). This led to rounding errors in some cases, thus the sum of percentages did not always add up to 100%. A further facet that needs consideration is that some reviews included multiple health outcomes at a time, hence they were mentioned repeatedly in the detailed results for PA, SB and HE. Additionally, the total number of included publications in the reviews has been used for the results which led to some studies being included two or three times. Between-group effects were indicated as temporary if significant differences between the groups were only present at one and not at all timepoints following the intervention. Effect measures from included meta-analyses were reported in greater detail than systematic reviews due to the additional information provided by the quantitative report and subgroup analyses. Standardized mean differences (SMD), also known as Cohen’s d, were classified with 0.2 as small, 0.5 as moderate, and 0.8 as large [45]. Hedge’s g was also interpreted by the same rule of thumb. Heterogeneity was reported using the *I*^*2*^ value, where values of 0 to 40% may indicate no important, 30 to 60% indicate moderate, 50 to 90% substantial, and 75 to 100% considerable heterogeneity [46]. Due to inconsistent reporting, additional values for significance of heterogeneity like Q and X^2^ were not reported.

## Results

Out of the 3895 reviews initially located and downloaded, 587 doublets were removed. During title and abstract screening, additional 3233 studies were excluded, with 75 studies remaining for full text screening. Sixty-four of these articles were excluded due to above mentioned exclusion criteria. This resulted in a total of 11 systematic reviews and meta-analyses which were included in this umbrella review [47–57] (for more details see Flow-Chart in Fig. 1). The updated search located 472 additional articles which were all excluded after title and abstract screening.

### Description of the included studies

The 11 reviews included a total of 195 publications (182 studies) published between 1998 and 2018, with 167 of these publications being included once throughout the reviews and 13 publications being included in two or three reviews, accounting for 28 publications.

The included original research studies were mainly conducted in USA and Canada and Europe, and the most common study designs were randomized control trials (RCTs). The duration of interventions ranged from one session to 24 months, with the majority (92%) of interventions lasting at least 4 weeks. Sample sizes ranged from 458 [51] to 73,417 participants [53] for the reviews and added up to 114,430 participants throughout all studies. The full details of study characteristics of articles included in the umbrella review are displayed in Table 1.

**Table 1:** Study characteristics of articles included in the umbrella review

Author type of review	Aim	Target population Setting	Country (number of studies)	N studies (N participants, age [years])	PA/SB/HE Main outcomes inclusion criteria (device-measured/ self-reported/ both)	Intervention effectiveness for PA/SB/HE	AMSTAR Review Quality
Böhm et al. 2019 [47] Systematic review	To examine mHealth effectiveness for PA	children and/or adolescents NA	Australia (2), New Zealand (1), Canada (1), Israel (1), Poland (1), USA (1)	7 (1164, 8–18)	**PA** Measured at least 1 PA-related variable as the outcome, (5/ 0/ 2)	**PA**: Ø	6/11 medium
Buckingham et al. 2019 [53] Systematic review	To examine mHealth effectiveness, feasibility and acceptability for PA and SB	NA workplace	USA (11), Australia (5), Canada (2), Netherlands (2), Belgium (1), Singapore (1), Finland (1), Norway (1), in multiple countries (1)	30 publications, 25 (73,417, 18+)	**PA/SB** Any quantitative measure as primary outcome (21/ 4/ 0)	**PA**: 14/25 (56%) ↑ over time or vs control 6/25 (24%) Ø 3/25 (12%) ↓ over time or vs control 2/25 (8%) N/A Between-group difference for ↑ of around 847 (95% CI 68–1625) to 2183 (95% CI 992–3344) steps/day **SB**: 4/10 (40%) ↓over time or vs control 3/10 (30%) Ø 3/10 (30%) ↑over time or vs control	7/11 medium
Direito et al. 2017 [52] Systematic review and meta-analysis	To examine mHealth effectiveness for PA and SB To investigate relationship between the effect size and the nature of PA/SB outcomes and to code the BCTs	NA NA	USA (11), Australia (3), United Kingdom (3), Austria (1), Portugal (1), Ireland (1), Canada (1)	21 (1701, 8.4–71.7)	**PA/SB** Duration or an estimate of energy expenditure (9/ 12/ 4) 1 not validated	**PA:** *Total PA* (7 studies): Ø SMD = 0.14, 95% CI − 0.12 - 0.41, I^2^ = 60% *MVPA* (9 studies):Ø SMD = 0.37, 95% CI − 0.03 - 0.77, I^2^ = 78% *Walking* (8 studies): Ø SMD = 0.14, 95%CI − 0.01 - 0.29, I^2^ = 0% **SB** (5 studies): ↓ SMD = − 0.26, 95% CI − 0.53 - − 0.00, I^2^ = 0%	8/11 medium
Ferrer et al. 2017 [51] Systematic review	To examine eHealth effectiveness for PA	NA Facebook	Not reported	8 (458, all ages (*M* = 24.3, *SD* = 7,5) 93,4% female	**PA** Either as a primary or a secondary outcome measure (2/ 4/ 2)	**PA:** 2/5 (40%) RCTs ↑ group by time interaction for steps per week and light PA participation 0/5 (0%) RCTs reported significant main effects for group,4/5 (80%) RCTs reported significant main effects for time 1/3 (33%) non-RCTs studies ↑ group by time interactions for self-reported total PA 1/3 (33%) ↑ total steps during the social condition 1/3 (33%) ↑ self-reported mean minutes per week for all categories of PA	4/11 medium
Hamel et al. 2011 [56] Systematic review	To examine eHealth effectiveness for PA and HE	preadolescents and adolescents, 8–18 years NA	USA (11), Belgium (3)	14 (6123 reported, 9–18)	**PA** PA or a PA-related health change as an outcome variable (2/ 10/ 2)	**PA:** 6/9 (67%) school-based interventions either ↑ PA in the intervention conditions and/or ↓ weight or BMI 2/5 (40%) home-based interventions either ↑ PA or ↓ BMI	4/11 medium
McIntosh et al. 2017 [50] Systematic review	To examine eHealth effectiveness for PA	young people attending school, college or university NA	USA (4), Netherlands (2), Thailand (1), Japan (1), Canada (1), Europe (1)	10 (5352, young people)	**PA** Primary or secondary outcome (8/ 1/ 1)	**PA:** 8/10 (80%) ↑ over time or vs control	5/11 medium
Muellemann et al. 2018 [49] Systematic review	To examine eHealth effectiveness for PA To compare effectiveness with either no intervention or a non-eHealth intervention.	older adults, 55 years or above NA	USA (11), Netherlands (3), Belgium (1), Spain (1), Australia (1), New Zealand (1), Malaysia (1)	25 publications, 20 (6671, 56–79.8 years)	**PA** Intervention effectiveness for any measure of PA (5/ 13/ 2)	**PA:** 9/9 (100%) web-based interventions ↑ over time or vs control 4/7 (57%) telephone-based interventions ↑ over time or vs control 3/4 (75%) text messaging-based interventions ↑ group over time or vs control	7/11 medium
Nour et al. 2016 [54] Systematic review and meta-analysis	To examine e/mHealth efficacy for HE	young adults, 18 to 35 years NA	USA (8), Australia (4), New Zealand (1), Malaysia (1)	14 (7984, *M* = 20.8)	**HE** Primary or secondary aim of increasing FVI (0/ 14/ 0), 5 not validated	**HE:** *FVI* (8 studies): ↑ SMD = 0.22 95% CI 0.11–0.33, I^2^ = 68.5% *Vegetable intake* (5 studies): ↑ SMD = 0.15 95% CI 0.04–0.28, I^2^ = 31.4%	9/11 high
Rocha et al. 2019 [55] Meta-analysis	To examine eHealth effectiveness for HE To investigate the relationship of effectiveness and intervention characteristics (eHealth tool, tailoring, BCTs, and age group)	NA NA	USA (10), Netherlands (3), Scotland (1), Belgium (1), Portugal (1), Italy (1), Sweden (1), New Zealand (1)	19 (6894, *M* = 4,5 to 57,75)	**HE** Reporting FVI results quantitatively (0/ 19/ 0), 5 not validated	**HE:** *FVI* (19 studies): ↑ *g =* 0.26 95% CI 0.17, 0.35, I^2^ = 62.77, *p* < .001	6/11 medium
Schoeppe et al. 2016 [57] Systematic review	To examine mHealth effectiveness for PA, SB and HE	children and/or adults NA	USA (9), Australia (6), Canada (3), Switzerland (2), Netherlands (2), Ireland (2), Italy (1), Israel (1), New Zealand (1)	30 publications, 27 (2699, 8–71)	**PA/SB/HE** Efficacy for behavior change. All types and units of measurements (8/ 13/ 6)	**PA SB and/or HE**: 19/27 (70%) ↑ in behavioral and related health outcomes either over time or vs control 5/10 (50%) ↑ single health behavior interventions vs control 7/17 (41%) ↑ multiple health behavior interventions vs control 8/13 (62%) ↑app in conjunction with other intervention strategies vs control 5/14 (36%) ↑ stand-alone app interventions vs control	4/11 medium
Stephenson et al. 2017 [48] Systematic review and meta-analysis	To examine e/mHealth for SB To identify the BCTs used within interventions	adults, 18 years or above NA	Not reported	17 (1967, *M* = 20,4 - 64,1)	**SB** Device-measured or self-reported or proxy measure of SB (8/ 6/ 3)	**SB** (15 studies): ↓ −41.28 min/day 95% CI −60.99 - − 21.58, I^2^ = 77% at end point follow-up	5/11 medium

*Abbreviations: HE* healthy eating, *M* mean, *NA* not available, *PA* physical activity, *RCT* randomized control trial, *SB* sedentary behavior, *SD* standard deviation, *USA* United States of America

Two reviews focused on children and adolescents [47, 56], four focused adults [48, 49, 53, 54] and five included participants of all ages [50–52, 55, 57]. Five systematic reviews focused on PA outcomes [47, 49–51, 56], one meta-analysis focused on SB outcomes [48], and one meta-analysis and one systematic review included both PA and SB outcomes [52, 53]. HE was the main outcome in two meta-analyses [54, 55], and one systematic review included PA, SB and HE as main outcomes [57].

Eight reviews reported the use of theoretical frameworks [47, 49–51, 53, 54, 56, 57], and 78/125 (62%) publications in these reviews reported the use of a theoretical foundation. The most common reported theories were social cognitive theory (*n* = 29), transtheoretical model (*n* = 16), theory of planned behavior (*n* = 10), self-determination theory (*n* = 10) and I-change model (*n* = 7). Four reviews [48, 52, 53, 55] coded the use of BCTs using a taxonomy of behavior change [20, 21] and two reviews [47, 51] reported BCTs without coding them. The BCTs, which were most frequently reported by the reviews, were goal setting (*n* = 5), self-monitoring (*n* = 4), social support (*n* = 4), prompts/cues (*n* = 4), feedback on the behavior (*n* = 3) and instruction on how to perform the behavior (*n* = 2). Since the BCTs were neither coded nor reported in a comparable way by the reviews, a more detailed summary was not feasible.

The majority of intervention studies were socially embedded (111/182, 62%). School, university or college settings were mentioned in 45 studies, workplace in 37 studies, home and/or community-based study populations were reported in 17 studies, while two studies reported a combination of workplace and home setting. A social media setting was mentioned in eight studies, and supermarket and online setting in one study each. Two reviews [56, 57] examined whether the interventions involved social support from the setting or solely took place in this context. Social support through peers and/or friendly challenges was described in six studies [57] and parental involvement in three studies [56]. None of the reviews reported about the use of JITAI or EMI.

### Overall effectiveness

The heterogeneity of the included studies concerning study type, outcome parameter, and assessment method was high. Thus, the overall effectiveness reported in the reviews is displayed in the following paragraph for any significant differences, which were found for the e/mHealth interventions over time or vs. a control group. Of all included studies, 10/182 did not report intervention effectiveness. The remaining 172 studies found a significant benefit for the intervention group over time and/or vs. a control group in 101/172 (59%) cases. No significant differences were found in 68/172 (40%) studies, and 3/172 (2%) resulted in a significant deterioration of the parameter over time and/or vs. control (see Table 1).

### Effectiveness vs. control

The between group differences for the included systematic reviews are displayed in the following chapters and the results of the included meta-analyses are reported in further detail.

### PA

PA (i.e. time spent in different PA intensities, step count, PA frequency, PA goal achievement, school related PA, and leisure time PA) was assessed by seven systematic reviews (PA outcome in 106 studies) [47, 49–51, 53, 56, 57] and one meta-analysis (PA outcome in 20 studies) [52]. Of the 126 studies included in these reviews, 58 studies used device-measured outcomes, 52 used self-report (1 not validated), and 16 used a combination of both measures.

Systematic reviews concerning PA did not report group differences or did not use a control group in 14/106 studies. The remaining 92 studies found significant group differences in favor of the intervention group in 19/92 (21%) studies, temporary significant group differences in favor of the intervention group in 25/92 (27%) studies and 49/92 (53%) showed no significant differences between the groups. One meta-analysis [52] included participants aged from 8.4 to 71.7 years and found no significant pooled effects using a random effect model between the eHealth and a usual/minimal care group for total PA (seven studies, SMD = 0.14, 95% CI [− 0.12, 0.41]; *Ι*^2^ = 60%), MVPA (nine studies, SMD = 0.37, 95% CI [− 0.03, 0.77]; *Ι*^2^ = 78%) and measures of walking (eight studies reporting steps/day and walking duration/day, SMD = 0.14, 95% CI [− 0.01, 0.29]; *I*^2^ = 0%). Subgroup analysis between device-measured and self-reported results showed no significant differences in the eHealth group for total PA, MVPA and walking.

### SB

SB (i.e. sitting time (overall and occupational), sedentary time (overall and occupational), screen time, and computer activity) was assessed by two systematic reviews (SB outcome in 13 studies) [48, 57] and two meta-analyses (SB outcome in 20 studies) [52, 53]. Of the 33 studies included in these reviews, 15 studies used device-measured outcomes, 16 used self-report (one not validated), and two used a combination of both measures.

The systematic reviews concerning SB included 4/13 studies which did not report group differences or did not involve a control group. The remaining nine studies showed a significant group difference in favor of the intervention group in 2/9 (22%) studies, 6/9 (67%) studies with no significant differences between the groups, and 1/9 (11%) reported a significant group difference in favor of the control group. The first meta-analysis (five studies) [52] which included participants aged from 8.4 to 71.7 years found a significant reduction of SB in favor of the intervention group using a random effect model. This pooled effect was negative and small (SMD = − 0.26, 95% CI [− 0.53, − 0.00]; *I*^2^ = 0%) with no evidence of heterogeneity. Subgroup analysis between device-measured and self-reported results showed no significant differences for the intervention group in SB. The second meta-analysis on SB (15 studies) [48] included only adults (20.4 to 64.1 years) and showed a significant pooled reduction of SB with a substantial heterogeneity (− 41.28 min/day, 95% CI [− 0.99, − 21.58], *I*^2^ = 77%; *n* = 1402) in favor of the intervention group at the end point follow-up measurement using a random effect model. Analysis for device-measured (eight studies) results showed a significant pooled reduction of − 35.07 min/day with a low heterogeneity (95% CI [− 46.57, − 23.57], *I*^2^ = 21%; *n* = 595), while self-reported measures (seven studies) led to a significant reduction of − 52.66 min/day with a considerable heterogeneity (95% CI, [− 93.63, − 11.69], *I*^2^ = 88%; *n* = 807) at end point. The comparison between device-measured and self-reported results has not been conducted by this meta-analysis. The additional analysis of short-term measures for overall SB (less than 3 months, 10 studies) showed a significant mean reduction of − 42.42 min/day with a substantial heterogeneity (95% CI [− 63.21, − 21.63], *I*^2^ = 61%; *n* = 760), the medium-term measures (3 to 6 months, five studies) showed a significant mean reduction of − 37.23 min/day with a considerable heterogeneity (95% CI [− 73.70, − 0.75], *I*^2^ = 85%; *n* = 691) and the long-term measures (over 6 months, three studies) showed no significant mean reduction with a low heterogeneity (− 1.65 min/day, 95% CI [− 14.77, 11.47], *I*^2^ = 23%; *n* = 670).

### HE

HE (i.e. FVI, vegetable intake, and healthy dietary choices) was assessed by one systematic review (HE outcome in 13 studies) [57] and two meta-analyses (HE outcome in 33 studies, focus on FVI) [54, 55]. All of the 46 studies included in these reviews used self-reported results, 10 of which were not validated.

The systematic review concerning HE did not report group differences or did not involve a control group for 1/13 studies. The remaining 12 studies found a significant group difference in favor of the intervention group in 2/12 (17%) studies, a temporary significant group difference in favor of the intervention group in 3/12 (25%) studies and 7/12 (58%) showed no significant differences between the groups. One meta-analysis [54] included young adults (M = 20.8 years) and showed a significant increase in FVI (eight studies) calculated by a random effect model with a small pooled Cohen’s *d* of 0.22 (95% CI [0.11, 0.33]) and a substantial heterogeneity (*I*^2^ = 68.5%). Effects for vegetable intake alone were also assessed (five studies) and the pooled effect showed a negligible effect with low heterogeneity (Cohen’s *d* = 0.15, 95% CI [0.04, 0.28], *I*^2^ = 31.4%). The second meta-analysis [55] included participants of all ages (4.5 to 57.75 years) and found a significant increase of FVI in favor of the intervention group using a random effect model with a small Hedge’s *g* and substantial between study heterogeneity (*g* = 0.26, SE = 0.05, 95% CI [0.17, 0.35], *I*^2^ = 62.77). Subgroup analyses revealed that computer-based (i.e. non-Internet based) eHealth interventions (three studies) showed the largest effect (*g* = 0.44), followed by SMS interventions (three studies) with a Hedge’s *g* of 0.41, while internet-based interventions (nine studies) showed a Hedge’s *g* of 0.19 and CD-ROM, mobile apps and video game interventions (four studies) showed no significant improvements. The subgroup analysis relating to age groups yielded no significant differences between adults (11 studies), adolescents (four studies) and children (four studies). Interventions including adults and adolescents showed significant improvements in favor of the intervention group with Hedge’s g of 0.26 and 0.35 respectively, while interventions conducted with children showed no significant effects.

### Determinants of effective interventions

The extraction of effect sizes regarding the influence of theoretical foundation/BCTs, social influences and EMI/JITAs on the efficiency of e/mHealth interventions was not feasible so that only descriptive results were reported in this umbrella review (see Table 2).

**Table 2:** Intervention effectiveness and the reported use of theoretical foundation, BCT, social context and EMI/JITAI in the included reviews

Author Type of review	Intervention duration Included study designs	Theoretical foundation	BCT	Social context	EMI/JITAI
Böhm et al. 2019 [47] Systematic review	1–12 months 4 RCTs, 1 RC cross over, 2 before-and-after trials	Total number of theory-based studies NA 4/7 (57%) social cognitive theory	3/7 (43%) additional BCTs	7/7 (100%) recruited in schools with interventions in and outside the school setting Combining school-based interventions with family or community involvement for social support is potentially effective	NA
Buckingham et al. 2019 [53] Systematic review	1–12 months 10 RCTs, 10 prospective cohort studies, 1 combination of methods mentioned above, 3 cluster-RCT, 1 parallel group uncontrolled randomized trial, 1 prospective cluster trial with an asynchronous control group	15/25 (60%) based on a named behavior change theory and/or principles of behavioral economics 2/25 (8%) studies alluded to BCTs or theory in their discussion 8/25 (32%) neither theory nor BCT	Most frequent BCTs: Self-monitoring of the behavior or outcome (*n* = 22, 88% of studies) provision of feedback on the behavior or outcome (*n* = 21, 84%) goal setting for the behavior or outcome (*n* = 17, 68%) social comparison (*n* = 14, 56%) social support (*n* = 12, 48%)	25/25 (100%) public and private sector workplace setting No associations were found between type of workplace and impact on PA	NA
Direito et al. 2017 [52] Systematic review and meta-analysis	Median = 9 weeks 21 RTCs	NA	Total BCTs: *M* = 5.4/93 BCTs (*SD* = 2.6, range 0 to 12) More BCTs were employed with intervention groups (*M* = 6.9, *SD* = 2.6, range 2 to 12) than with comparator groups (*M* = 3.1, *SD* = 2.2, range 0 to 10) Most frequent BCTs: 81% goal setting (behavior 74% self-monitoring of behavior 65% social support (unspecified) 55% feedback on behavior 55% instruction on how to perform the behavior 48% adding objects to the environment 45% information about health consequences 45% prompts/cues	NA	NA Prompts and cues common BCT
Ferrer et al. 2017 [51] Systematic review	3–12 weeks 5 RCTs, 2 single group designs, 1 within-subject crossover	4/8 (50%) theory-based interventions 1/8 (13%) theory of planned behavior 1/8 (13%) social cognitive theory 2/8 (25%) not specified	8/8 (100%) interventions used behavior modification strategies including goal setting, self-monitoring, prompts, and social support	8/8 (100%) social-media interventions (facebook) 7/8 (88%) of the Facebook interventions ↑ some type of PA behavior change (i.e., interactions, main effects for time, differences between conditions) 2/8 (25%) ↑ for the treatment group compared to the control group.	NA
Hamel et al. 2011 [56] Systematic review	2 weeks - 2 years 7 RCTs, 5 quasi-experimental, 1 repeated measures, 1 pretest-posttest control group design	9/14 (64%) theory-based interventions Social cognitive theory most frequent Other theories: Theory of reasoned action transtheoretical model health belief model theory of planned behavior attitude, social influence, and self-efficacy model 4/14 (29%) having more than one theory	NA	9/14 (64%) school-based 3/14 (21%) home-based 1/14 (7%) camp- and home-based 1/14 (7%) scout troop and home-based 3/14 (21%) of these included parental involvement School-based interventions were more effective than e.g. home-based interventions and parental support might be important	NA
McIntosh et al. 2017 [50] Systematic review	6 weeks - 4 months 6 RCTs, 3 before and after quasi-experimental designs 1 cluster-RCT	9/10 (90%) theory-based interventions 5/10 (50%) social cognitive theory 2/10 (20%) theory of planned behavior 1/10 (10%) transtheoretical model 1/10 (10%) SMART goals	NA	10/10 (100%) students attending school, college or university Effect of social context not analyzed	NA
Muellemann et al. 2018 [49] Systematic review	1–24 months 18 RCTs, 2 quasi-experimental design	16/20 (80%) theory-based interventions most common: 9/20 (45%) social cognitive theory 8/20 (40%) transtheoretical model 7/20 (35%) self-determination theory 7/20 (35%) i-change model	NA	NA	NA
Nour et al. 2016 [54] Systematic review and meta-analysis	one-off contact - 6 months 14 RCTs	6/14 (43%) theory- or education-based interventions 5/14 (36%) transtheoretical model 2/14 (14%) social cognitive theory 2/14 (14%) theory of planned behavior and the theory of habit formation 7/14 (50%) applied self-efficacy in their intervention	NA	no group intervention 11/14 (79%) university setting 2/14 (14%) N.A. 1/14 (7%) home based Effect of social context not analyzed	NA
Rocha et al. 2019 [55] Meta-analysis	one-time session - 24 weeks 14 RCTs, 3 cluster-RCTs, 2 non-randomized studies	NA	1 to 7 BCTs used20/40 BCT categories identified Most common:68% provide instruction on how to perform the behavior 47% Provide feedback on performance 26% goal setting on behavior	8/19 (42%) school setting 6/19 (32%) community-based 2/19 (11%) workplace-based 1/19 (5%) clinic-based (prevention) 1/19 (5%) online-based 1/19 (5%) supermarket-based Effect of social context not analyzed	NA 15/19 (79%) tailored interventions 4/19 (21%) were nontailored interventions
Schoeppe et al. 2016 [57] Systematic review	1–24 weeks 19 RCTs, 4 pre-post studies, 3 controlled trials, 1 randomized trial	15/27 (56%) theory-based interventions 4/27 (15%) transtheoretical model 4/27 (15%) social cognitive theory 3/27 (11%) self-determination theory 2/27 (7%) theory of planned behavior 1/27 (4%) control systems 1/27 (4%) theory of self-regulation 1/27 (4%) behavior change wheel	NA	6/27 (22%) social support 3/6 interaction with peers 4/6 friendly team challenges Effect of social context not analyzed	NA
Stephenson et al. 2017 [48] Systematic review and meta-analysis	5 days - 24 months 17 RCTs	NA	1 to 15 BCTs used 20/93 BCTs categories identified most common: 88% instruction on how to perform a behavior 71% social support (unspecified) 65% prompts and cues 65% adding objects to the environment	10/17 (59%) workplace setting 5/17 (29%) Community/home setting 2/17 (12%) workplace and community/home setting Effect of social context not analyzed	NA Prompts and cues common BCT

*Abbreviations: BCT* behavior change technique, *CI* confidence interval, *HE* healthy eating, *EMI* ecological momentary intervention, *FVI* fruit and vegetable intake, *g* Hedges’ g, *I*^*2*^ percentage of variation across studies that is due to heterogeneity rather than chance, *JITAI* just-in-time adaptive intervention, *M* mean, *NA* not available *PA* physical activity, *RCT* randomized control trial, *SB* sedentary behavior, *SMD* standardized mean difference, ↑ significant higher value (*p* < 0.05), Ø no significant difference, ↓ significant lower value

Theoretical foundation and BCTs were mentioned in all the included reviews [47–57]. One review [50] related to PA noted that 5/5 (100%) studies based on social cognitive theory led to significant differences over time or vs. control compared to 1/2 (50%) for theory of planned behavior and 1/1 (100%) showing a temporary significant difference directly after the intervention for transtheoretical model. Another review concerning PA [49] also found theory-based interventions more effective than those without a theoretical foundation. A third review concerning PA [56], which found that 6/9 (67%) theory-based interventions showed significant improvements of the intervention group over time or vs. control, while only 2/5 (40%) without a theoretical foundation led to such improvements, is in line with these findings. The inclusion of BCTs was associated with higher effectiveness of PA, SB and HE interventions in one review [57]. However, the question which BCTs are linked to effectiveness has not been answered by this review. Two meta-analyses [48, 52] reported the usage of BCTs for PA and SB interventions, but did not link the use of BCTs to effectiveness due to the small number of studies included. For healthy eating behavior, the use of BCTs was one key component of successful interventions, while the impact of using multiple BCTs remained unclear [54]. Further, a more recent meta-analysis [55] revealed that the inclusion of seven to eight BCTs (four studies) resulted in a statistically significant larger effect size (SMD = 0.42, SE = 0.10, 95% CI [0.21, 0.62], *p* < .001) than those involving four to six BCTs (seven studies) and one to three BCTs (seven studies). In a next step, the meta-analysis found no statistically evidence for specific BCTs yielding larger effect sizes.

The influence of a social settings concerning effectiveness has not been reported in detail by the included reviews and two reviews [56, 57] reported on the matter at all. The integration of eHealth interventions in school settings was reported to lead more often (6/9, 67%) to positive effects on PA or weight reduction in comparison to home-based interventions (2/5, 40%) [56]. Another possible influence on effectiveness mentioned in this review was parental influence [56]. The second review about mHealth interventions points out that efficient interventions often include social support related to peers and friendly team challenges among many other facets [57]. However, since both reviews did not report effect sizes, and there were a variety of other possible facets contributing to effectiveness, the magnitude of the potential influence for social settings remains unclear.

Since none of the reviews reported the use of EMI/JITAIs, the question concerning their effectiveness has to be left unanswered by this umbrella review.

### Study quality

Mean study quality of the included reviews as assessed by the AMSTAR tool [44] (maximum score 11, score ratings: low = 0–3, medium = 4–7 and high = 8–11 [58]) was medium (M = 5,9/11) while one review scored high (9/11) [54]. None of the included reviews reported the conflict of interest of the included studies and only one review provided a list of all included and excluded studies [54]. For the score of every criterion see Additional file 2. Risk of bias ratings conducted by the authors of the included reviews was mainly medium to high with some studies of low risk (see Table 3).

**Table 3:** Time period, Intervention tools, quality of included studies in the reviews, and recommendations for future research

Author type of review	Time Period Searched (included studies)	mHealth/eHealth tools	Quality of included studies	Recommendations for future research
Böhm et al. 2019 [47] Systematic review	January 2012 to June 2018 (2014–2016)	Mobile phones, smartphones, tablets, or wearables	Tool: Cochrane Handbook for Systematic Reviews of Interventions Risk of bias: 2/5 (40%) medium 3/5 (60%) high	1) PA intervention programs for children/adolescents with a greater BMI z-score 2) intervention programs with a longer period of time (≥6 months) 3) sufficiently large number of participants (≥250) 4) bypass self-reported measurements 5) implement theoretical frameworks and BCTs 6) follow-up beyond postintervention 7) age- and sex-specific interventions 8) engagement of children and adolescents with wearable activity trackers 9) impact of social support (school/family) 10) multicomponent interventions 11) cost-effectiveness analyses
Buckingham et al. 2019 [53] Systematic review	January 2007 to February 2018 (2009–2018)	mHealth interventions: mobile phone, smartphone apps, personal digital assistants, tablets, wearable activity monitors/ trackers	Tool: Effective Public Health Practice Project Quality rating: 1/25 (4%) strong, 9/25 (36%) moderate, 15/25 (60%) weak	1) larger samples and more diverse workspace settings 2) report intervention components and outcomes in greater detail 3) SB in addition to PA, and bypass self-report 4) no-intervention control or a reliable baseline measurement 5) wider impact on health and wellbeing 6) mixed and qualitative methods 7) adverse events associated with mHealth use 8) mHealth vs multi-component interventions 9) subgroup differences
Direito et al. 2017 [52] Systematic review and Meta-Analysis of RCTs	From earliest availableto January 2015 (2007–2014)	mHealth interventions: mobile devices, such as mobile phones, patient monitoring devices, personal digital assistants	Tool: Cochrane Collaboration’s tool No total rating: High Risk of Bias for blinding, unclear allocation, other biases were low for most studies	1) long-term effectiveness and cost-effectiveness of mHealth interventions 2) dose-response relationship between intervention exposure and outcomes 3) report intervention components and outcomes in greater detail 4) efficacy of more advanced technology than SMS
Ferrer et al. 2017 [51] Systematic review	not specified (2010–2014)	Facebook based interventions	Not assessed	1) no-intervention control 2) target a broader diversity of participants 3) attrition rates for varying durations of interventions 4) theory-based content and measure the effects of those mediators 5) effectivity of social support 6) validate self-report measures against device-measured outcomes of PA 7) match the PA assessment method to the stated goals and outcomes of the intervention 8) long term follow-up
Hamel et al. 2011 [56] Systematic review	1998 to 2010 (1999–2009)	Computer- and web-based interventions	Tool: Critical Appraisal Skills Programme of the Public Health Resource Unit Quality rating: No summary presented	1) bypass self-report 2) sex specific interventions 3) involve support persons (e.g. parents or peers) and analyze effectivity 4) integrate into existing school curriculum 5) include a theoretical framework 6) individual tailoring
McIntosh et al. 2017 [50] Systematic review	2010 to July 2016 (2010–2014)	Web-based or eHealth interventions	Tool: based on the critical appraisal for public health checklist Quality rating: 3/10 (30%) high 7/10 (70%) moderate	1) longer follow-up 2) address bias incorporated with self-reporting methods 3) utilize theoretical foundation for eHealth interventions 4) relationship of confounding facets to effectiveness 5) conduct power analysis of studies 6) scale up interventions
Muellemann et al. 2018 [49] Systematic review	from earliest available to April 2017 (1997–2017)	eHealth interventions: computer, telephone smartphone, or tablet	Tool: Cochrane Collaboration’s tool for assessing risk of bias Risk of bias: 1/20 (95%), low 19/20 (95%) moderate to high	1) eHealth interventions vs non-eHealth interventions promoting PA in older adults
Nour et al. 2016 [54] Systematic review and Meta-Analysis	1990 to August 2015 (2007–2014)	eHealth- and mHealth-based interventions: texting, email, mobile phone apps, phone calls, or websites	Tool: Cochrane Collaboration’s tool for assessing risk of bias Risk of bias rating: majority of the studies unclear to high risk (attrition bias) 2/14 (14%) studies additionally high detection bias	1) longer follow-up in intervention 2) secondary outcomes (e.g.) weight and indicators of cardiovascular health) 3) focus primarily on vegetables 4) combine efficacious strategies and repeat exposure at a later date 5) develop validated tools for measuring vegetable intake in young adults 6) quantify a serving of vegetables 7) implement Biomarkers (e.g. vitamin C and beta-carotene) 8) more diverse samples 9) cost effectiveness for upscaling interventions 10) conduct process evaluations
Rocha et al. 2019 [55] Meta-Analysis	1999 to July 2018 (1999–2017)	eHealth interventions: mobile devices (apps, text messages via cellphone), web or internet-based programs, computer-based programs (non-Internet based), and video games.	Tool: guided by the Cochrane’s Risk of Bias Tool for RCTs Quality rating: 5/19 (26%) good 12/19 (63%) fair 2/19 (11%) poor	1) tailor based on distal correlates and proximal determinants of dietary habits 2) link the types of BCTs implemented in the eHealth interventions to effectiveness 3) develop validated tools for measuring FVI 4) report intervention components and outcomes in greater detail 5) use of the CALO-RE taxonomy for uniformity in the reporting of BCTs
Schoeppe et al. 2016 [57] Systematic review	January 2006 to November 2016 (2010–2016)	mHealth (App interventions): stand-alone intervention using apps only, or a multi-component intervention including apps	Tool: 25-point criteria adapted from the CONSORT checklists Quality rating: 11/27 (40%) high 8/27 (30%) fair 8/27 (30%) low	1) test the efficacy of specific app features and BCTs 2) efficacy of stand-alone app intervention vs multi-component app interventions 3) efficacy of app vs website, print-based and face-to-face interventions 4) utilize larger sample sizes 5) tailor app interventions to specific population groups with high app usage (e.g., women, young people) 6) report app usage statistics using device and self-report measures 7) optimal duration and intensity of app interventions 8) user engagement and retention in app interventions 9) relationship between user engagement and intervention efficacy (considering socio-demographic and psychosocial facets)
Stephenson et al. 2017 [48] Systematic Review and Meta-analysis	from earliest available to June 2016 (2012–2016)	Computer, mobile or wearable technology	Tool: Cochrane Collaboration’s risk of bias tool Risk of bias: 1/17 (6%) low 3/17 (18%) unclear 13/17 (76%) high	1) focus on attrition rates 2) improve reporting of BCTs 3) improve detection bias by using objective measurement tools of SB 4) conduct extended follow-up 5) include outcome measures that will be of interest to workplaces and policy makers 6) use adaptive interventions

*Abbreviations: AMSTAR* assessment of multiple systematic reviews, *App* smartphone application, *BCT* behavior change technique, *CONSORT* consolidated standards of reporting trials, *eHealth* electronic health, *FYI* fruit and vegetable intake, *mHealth* mobile health, *PA* physical activity, *SB* sedentary behavior

## Discussion

This umbrella review provided an overview of e/mHealth interventions concerning PA, SB and HE for primary prevention with a special focus on potentially important facets and their contribution to intervention effectiveness. To avoid an overwhelming heterogeneity in the included reviews, these facets have been pre-defined based on the current literature and previous umbrella reviews as theoretical foundation, BCTs, social context and the use of JITAI/EMI. To the best of our knowledge, this umbrella review is the first to systematically analyze the potential impact for those predefined facets.

### Effectiveness of e/mHealth interventions

Overall, findings of this umbrella review suggested that a majority (59%) of e/mHealth interventions were effective (including interventions eliciting short-term effects and interventions without control-group comparison). Since multiple studies reported a high heterogeneity (with low to high quality ratings), this result has to be interpreted with caution. Results of the systematic reviews including a control-group indicated that PA interventions were more often effective (48%) than interventions concerning HE (42%) and SB (22%). However, more than 50% of these effects for PA and HE interventions were only temporary and one SB study outcome (11%) was even in favor of the control group. In contrast to systematic reviews, quantitative findings of the included meta-analyses did not indicate any significant benefit for PA while SB and HE interventions showed significant small effects. A reason for the lack of effectiveness in the only meta-analysis concerning PA [52] may be that solely one original research study included a true control group and e/mHealth to usual/minimal care. Furthermore, the post hoc exploratory sensitivity analysis displayed two of the included studies as being the main reason for heterogeneity in this meta-analysis. After removal of these studies, results indicated a small but significant effect for MVPA (but none for total PA) and thereby partially support the findings of the systematic reviews that e/mHealth interventions can be effective tools to change all three health behaviors.

One facet which could have influenced the results of this umbrella review is the use of different assessment methods in the studies, as self-report measures are commonly reported to overestimate PA compared to sensor-based PA [59]. Considering the fact that some studies even used non-validated self-report tools in PA, SB and HE interventions, these facets could have highly influenced findings. In the present umbrella review, the comparison of self-reported and device-measured outcomes showed no significant differences for PA and SB in one meta-analysis [52], and another meta-analysis reported lower heterogeneity and a descriptive difference of SB reduction for device-measured results [48]. While all other reviews reported on the use of self-reported and device-measured results, the examination of influence on effectiveness has not been conducted and thus no assumptions about a potential impact of the measuring method could be made. However, examining the impact of the measurement method could be important, since self-reported and device-measured results often differ concerning the construct (e.g. measuring habitual PA or sport related PA) and the time epoch (e.g. regular/last week/month PA recall via questionnaire or measured PA during a defined time via accelerometry) [59]. Furthermore, the earliest study included in the reviews was published in 1997 and the complexity and capacity of sensors evolved rapidly since that time [60], allowing for more precise measurements and the combination of PA data with physiological parameters like heartrate or blood sugar [61]. This potential influence of different sensors on intervention effectiveness however, has not been considered in the reviews. Future reviews should specifically compare results derived by self-reports to device-measured outcomes and assess the impact of the complexity of sensors in order to further investigate the true impact of assessment methods and ease the interpretation of results.

The sustainability of intervention effects was reported to be low in the reviews for PA and HE, and quantification of one meta-analysis [48] showed that the effects of SB interventions diminish after 6 months, which is in accordance with other research [18]. Intervention duration and engagement are also important facets influencing intervention effectiveness [62], but the influence remains unclear due to a lack of reporting by the included reviews. Future reviews should consider this link, especially if they are comparing sustainability of intervention effects over time.

The use of eHealth compared to mHealth might also influence the effectiveness. However, results are inconclusive since most reviews did only assess the intervention type but not compare the impact on effectiveness. One meta-analysis [52] which quantified the results found computer-based interventions to cause superior effectiveness compared to web- and app-based interventions. However, since mHealth is a more recent development and the amount of evidence is limited, this trend might be modified with more sophisticated approaches and more study results in the future [63]. There is a clear need to include the comparison of effectiveness across devices in PA and SB interventions along with the influence of the age of participants in order to enhance and specify future interventions.

### Influence of theoretical foundation, BCT, social aspects and JITAIs on effectiveness

The diversity of results supported the importance to consider the underlying mechanisms for effective e/mHealth interventions in order to further develop the field of digital behavior change in general and in the area of primary prevention in particular.

The use of BCTs as a sub-section of theoretical foundation provided the most distinct picture and was highly associated with effective interventions for PA, SB and HE interventions in one systematic review [57]. This finding was further supported by the two meta-analyses concerning HE [54, 55]. The use of more BCTs enhanced intervention effectiveness for HE, whereas the impact of specific BCTs or combinations of BCTs remains unknown [55], which has been a common finding in reviews on HE interventions [64]. The meta-analyses concerning PA and SB did not report the impact of BCTs on effectiveness which should be addressed by future research. Support for the use of a theoretical foundation for effective e/mHealth interventions concerning PA was found in three reviews [49, 50, 56], and there were indications that social cognitive theory might be especially effective [50]. The overall higher effectiveness for theoretical founded interventions supported the findings of a previous review about internet interventions (not focused on primary prevention) [23] but in contrast to our results, the theory of planned behavior was found to be more effective than social cognitive theory. Since direct comparisons of theory vs. no theory in the included reviews were scarce and only descriptive, there is a need for further investigation and better documentation of theoretical backgrounds in intervention studies in order to draw a clear conclusion. The lack of reporting regarding the impact of theoretical foundation on effectiveness for SB and HE should additionally be addressed by further research. A further aspect to consider in future studies is the compatibility of static behavior change theories to the technological advances which has not been addressed by the included reviews. While dynamically changing theories like the adapted versions of the Theory of Planned Behavior [65] or the Social Cognitive Theory [66] has been promoted in the development of JITAIs [31] the impact on intervention effectiveness should be assessed in future interventions.

In contrast to the potential impact of theoretical foundation and BCTs, most reviews did neither report nor analyze the association of embedding interventions into social contexts (e.g. involving family, peers or co-workers in the intervention) and intervention effectiveness. Only three reviews [51, 56, 57] reported on that matter, but were unable to specify the influence due to a small sample and/or multiple other parameters linked to effectiveness. The importance of getting a better impression of social influences should however not be underestimated in order to conduct effective interventions in the future [67]. Including social facets can have an essential influence on intervention effectiveness and should be considered in future research [68]. Furthermore, intervention designs comparing e/mHealth interventions with clearly defined and controlled social contexts (e.g. social comparison, cooperative approaches) might help to gain evidence on the impact of social context.

No mention at all was found for the use of EMI/JITAI in this umbrella review. With the possibility to tailor and to continually adapt interventions to each person’s needs, as well as to deliver support at the most promising moment, there is a clear need for examination of this important field in the future [28–30].

### Strengths and limitations

The main strengths of this umbrella review consisted in summarizing the knowledge about the impact of multiple facets of effective behavior change interventions, derived from current literature, on effectiveness. Following a pre-registered protocol and systematically summarizing the evidence on the effectiveness of e/mHealth interventions in primary prevention ensured a replicable approach. Using a systematic search with pre-defined terms, following the PRISMA guidelines for reporting and using AMSTAR for quality assessment thereby enhanced the transparency of the results. The inclusion of systematic reviews and meta-analyses following PRISMA guidelines ensured a solid foundation of higher quality reviews and a systematic reporting of the original research results.

Nonetheless, there are several limitations concerning the current umbrella review that need to be considered. First, the results of this umbrella review highly depended on the detailed reporting of the desired parameters in the reviews. Even if the original research studies reported on the issue but the reviews did not, the result has not been considered for this umbrella review. Even though the included reviews had to follow the PRISMA statement themselves, the quality of reviews was medium with a high discrepancy of included original research studies ranging from low to high scores and including several non-RCT studies. This might have impacted the conclusions of this umbrella review as well. The fact that 13 publications were included twice or more might also bias the evidence since those studies get a higher impact on the overall results. Finally, important studies might not be included in any review article yet since the conduction and publication of reviews produces a certain time lag compared to the present evidence.

### Implications for practice and research

Results of this umbrella review can serve as a theoretical basis to conduct both, original research and review articles in the field of primary prevention using e/mHealth. Researchers should address the main research gaps, namely the impact of different theoretical foundations for interventions in different contexts, the adequate amount and types of BCTs, the impact of social context and enhancing interventions with JITAIs, by conducting original research studies or especially focused reviews to close research gaps. For practitioners, we recommend to implement theoretical foundation and BCTs to their e/mHealth interventions in order to enhance intervention effectiveness. Furthermore, e/mHealth interventions should be adapted once further evidence emerges in order to maximize the usefulness of this fast-changing field of behavior change.

### Future directions

Even though the included reviews were conducted over the course of nearly a decade and thus represent different stages of e/mHealth tools, recommendations for future research given by the authors of the included reviews have a lot in common (for more details see Table 3). Based on these recommendations, a clear need for PA and SB studies is stated to bypass self-report and use validated and comparable device-measured outcomes instead [47, 48, 50, 51, 53–56]. Mainly including device-measured outcomes will lead to a more comprehensive picture of intervention effectiveness even though other challenges arise from that approach (e.g. comparison of different epoch lengths [69]). The most promising aspect of device-measured outcomes and accelerometry in particular is the assessment of valid PA and SB data in real-time, resulting in a variety of outcome parameters which have the potential to be easily compared throughout different studies [60]. While device-measured assessments for HE are rarely used (but becoming more and more available [70]), HE interventions should only include validated tools and be aware of the advantages of each assessment to ensure the quality of results [71].

In order to analyze the influences of different intervention aspects on effectiveness, a uniform and full reporting of the intervention components (theoretical foundation, BCTs, social aspects, etc.), methods and outcomes is needed [48, 51–53, 55, 57]. Additionally, an exploration of the adequate dose and length of interventions [47, 48, 51, 52, 54, 57], the influence of social support [47, 50, 51, 56, 57] as well as individual tailoring (e.g. using JITAIs to deliver sex-, age- or BMI-specific interventions adapting to personal preferences) [47, 55–57] is needed for a better understanding of the determinants of effectiveness. Here, machine learning principles can enhance intervention effectiveness by allowing a highly personalized adaptation to the users’ needs and environmental requirements [31]. Future e/mHealth studies for behavior change should also conduct a priori power analyzes to include appropriate sample sizes in order to enhance the value of the results [47, 50, 53, 57] and assess cost-effectiveness [47, 52, 54].

## Conclusions

In summary, e/mHealth interventions can be effective tools for primary prevention in behavior change of PA, SB and HE, but the evidence for effectiveness is still limited. Theoretical foundation and the use of BCTs are promising determinants of effectiveness. However, there is still a research gap which theory and which BCTs are the most promising for primary prevention and for the inclusion of social contexts, JITAIs and other facets like the optimal dose and length of interventions. Therefore, future studies should limit methodological issues (e.g. non-validated tools) and use appropriate assessments (depending on the outcome variable of choice), and a more comprehensive and standardized way of reporting. In doing so, the benefit of the main advantages of e/mHealth, namely the large coverage, potential cost effectiveness and high adaptability to individual preferences and environmental facets, can be utilized to enhance behavior change in primary prevention.

## Supplementary information

**Additional file 1.** Detailed search history for all databases.

**Additional file 2.** AMSTAR score of every criterion.

## Data Availability

All relevant data output can be found in supplementary material online. If readers have further questions, further material can be made available upon request.

## Abbreviations

AMSTAR: Assessment of multiple systematic reviews
CI: Confidence interval
eHealth: Electronic health
EMI: Ecological momentary intervention
HE: Healthy eating
FVI: Fruit and vegetable intake
JITAIs: Just-in-time adaptive interventions
mHealth: Mobile health
min: Minutes
MVPA: Moderate to vigorous physical activity
PA: Physical activity
PRISMA: Preferred Reporting Items for Systematic Reviews and Meta-Analyses
PROSPERO: International prospective register of systematic reviews
RCT: Randomized control trial
SB: Sedentary behavior
SMD: Standardized mean differences
