# Supplementary material for: Key facets to build up eHealth and mHealth interventions to enhance physical activity, sedentary behavior and nutrition in healthy subjects – an umbrella review
Source: BMC Public Health. 2020 Oct 23;20:1605. doi: 10.1186/s12889-020-09700-7 (PMC7585171; doi:10.1186/s12889-020-09700-7)
Supplement: Supplementary file 1 — Additional file 1. Detailed search history for all databases. [file 12889_2020_9700_MOESM1_ESM.docx]

**PubMed search**

(((("Wireless Technology"[Mesh] OR "Telemedicine"[Mesh] OR mHealth OR mobile health OR "Smartphone"[Mesh] OR "Mobile Applications"[Mesh] OR "Text Messaging"[Mesh] OR "Internet"[Mesh] OR "Electronic Mail"[Mesh] OR mobile device OR web-based OR "Feedback, Sensory"[Mesh] OR "Wearable Electronic Devices"[Mesh] OR "Fitness Trackers"[Mesh] OR Acceleromet* OR Pedometer OR tablet computer* OR activity monitor*)

AND

("Exercise"[Mesh] OR "Movement"[Mesh] OR "Locomotion"[Mesh] OR "Sedentary Behavior"[Mesh] OR "Healthy Lifestyle"[Mesh] OR "Motor Activity"[Mesh] OR "Primary Prevention"[Mesh] OR "Preventive Health Services"[Mesh] OR "Physical Fitness"[Mesh] OR Physical Activity OR sport OR sitting OR weight loss OR "Diet"[Mesh] OR "Diet, Food, and Nutrition"[Mesh] OR "Snacks"[Mesh] OR "Food"[Mesh] OR "Healthy Diet"[Mesh] OR "Fruit"[Mesh] OR "Vegetables"[Mesh] OR "Meals"[Mesh] OR eating OR food consumption OR eating habits)

AND

("health behavior"[MeSH Terms] OR "Behavior"[Mesh] OR "Behavior and Behavior Mechanisms"[Mesh] OR Behavi* AND Change OR Behavi* AND Change Technique OR "Family"[Mesh] OR "Psychology, Social"[Mesh] OR "Friends"[Mesh] OR "Peer Group"[Mesh] OR "Peer Influence"[Mesh] OR "Schools"[Mesh] OR "Social Media"[Mesh] OR "Social Support"[Mesh] OR "Social Environment"[Mesh] OR "Social Networking"[Mesh] OR social OR "Ecological Momentary Assessment"[Mesh] OR "Precipitating Factors"[Mesh] OR Ecological Momentary Intervention OR EMI OR EMA OR Prompt* OR Just-in-Time OR trigger)))

AND

Review[ptyp])

Filters: Review; Publication date from 1990/01/01

**Scopus search**

( TITLE-ABS-KEY ( *mhealth*  OR  *"mobile health"*  OR  *telemedicine*  OR  *smartphone*  OR  *"text message"*  OR  *web-based*  OR  *wearable*  AND  *device*  OR  *accelermete**  OR  *pedomet**  OR  *internet*  OR  *e-mail*  OR  *"fitness tracker"* )  AND  TITLE-ABS-KEY ( *exercise*  OR  *"physical activity"*  OR  *movement*  OR  *sedentary*  OR  *fitness*  OR  *"health promotion"*  OR  *"active living"*  OR  *healthy*  AND  *livestyle*  OR  *activity*  OR  *diet*  OR  *nutrition*  OR  *food*  OR  *eating*  OR  *meals*  OR  *fruit*  OR  *vegetable*  OR  *"healthy eating"* )  AND  TITLE-ABS-KEY ( *family*  OR  *social*  OR  *school*  OR  *job*  OR  *work*  OR  *peer-group*  OR  *social*  AND  *media*  OR  *"social support "*  OR  *bct*  OR  *behavio**  OR  *"behavio* modification"*  OR  *"behavio* change"*  OR  *"behavior change technique"*  OR  *"ecological momentary intervention"*  OR  *"ecological momentary assessment"*  OR  *just-in-time*  OR  *ema*  OR  *emi*  OR  *prompting* ) )  AND  DOCTYPE ( *re* )

**Cochrane**

ID Search Hits

#1 MeSH descriptor: [Telemedicine] explode all trees 2153

#2 MeSH descriptor: [Mobile Applications] explode all trees 420

#3 MeSH descriptor: [Internet] explode all trees 3586

#4 MeSH descriptor: [Text Messaging] explode all trees 664

#5 MeSH descriptor: [Wireless Technology] explode all trees 34

#6 (mHealth):ti,ab,kw 771

#7 (mobile health):ti,ab,kw 3565

#8 MeSH descriptor: [Wearable Electronic Devices] explode all trees 329

#9 MeSH descriptor: [Fitness Trackers] explode all trees 53

#10 activity monitor 2995

#11 MeSH descriptor: [Wearable Electronic Devices] explode all trees 329

#12 MeSH descriptor: [Accelerometry] explode all trees 798

#13 #1 OR #2 OR #3 OR #4 OR #5 OR #6 OR #7 #8 OR #9 OR #10 OR #11 OR #12 10543

#14 MeSH descriptor: [Exercise] explode all trees 22235

#15 MeSH descriptor: [Healthy Lifestyle] explode all trees 456

#16 MeSH descriptor: [Health Promotion] explode all trees 5858

#17 MeSH descriptor: [Movement] explode all trees 29628

#18 MeSH descriptor: [Sedentary Behavior] explode all trees 965

#19 MeSH descriptor: [Physical Fitness] explode all trees 2942

#20 MeSH descriptor: [Locomotion] explode all trees 7724

#21 MeSH descriptor: [Sitting Position] explode all trees 25

#22 MeSH descriptor: [Preventive Health Services] explode all trees 29476

#23 MeSH descriptor: [Diet] explode all trees 17252

#24 MeSH descriptor: [Diet, Food, and Nutrition] explode all trees 49673

#25 MeSH descriptor: [Healthy Diet] explode all trees 300

#26 MeSH descriptor: [Fruit] explode all trees 2485

#27 MeSH descriptor: [Vegetables] explode all trees 1779

#28 MeSH descriptor: [Meals] explode all trees 1151

#29 MeSH descriptor: [Eating] explode all trees 3382

#30 MeSH descriptor: [Feeding Behavior] explode all trees 8313

#31 #14 OR #15 OR #16 OR #17 OR #18 #19 OR #20 OR #21 OR #22 OR #23 OR #24 OR #25 OR #26 OR #27 OR #28 OR #29 OR #30 97617

#32 MeSH descriptor: [Family] explode all trees 8514

#33 MeSH descriptor: [Psychology, Social] explode all trees 21927

#34 MeSH descriptor: [Friends] explode all trees 126

#35 MeSH descriptor: [Peer Group] explode all trees 1289

#36 MeSH descriptor: [Peer Influence] explode all trees 25

#37 MeSH descriptor: [Schools] explode all trees 2749

#38 MeSH descriptor: [Social Media] explode all trees 108

#39 MeSH descriptor: [Social Support] explode all trees 3181

#40 MeSH descriptor: [Social Environment] explode all trees 4111

#41 MeSH descriptor: [Social Networking] explode all trees 76

#42 (BCT):ti,ab,kw (Word variations have been searched) 414

#43 MeSH descriptor: [Behaviorism] explode all trees 2

#44 ("behavior change theory"):ti,ab,kw (Word variations have been searched) 109

#45 (behavio* AND change):ti,ab,kw (Word variations have been searched) 33260

#46 MeSH descriptor: [Behavior] explode all trees 83395

#47 MeSH descriptor: [Ecological Momentary Assessment] explode all trees 19

#48 ("just-in-time"):ti,ab,kw (Word variations have been searched) 100

#49 (ecological momentary assessment):ti,ab,kw (Word variations have been searched) 303

#50 (ecological momentary intervention):ti,ab,kw (Word variations have been searched) 172

#51 (trigger):ti,ab,kw (Word variations have been searched) 8348

#52 (prompting):ti,ab,kw (Word variations have been searched) 6869

#53 #32 OR #33 OR #34 OR #35 OR #36 OR #37 OR #38 OR #39 OR #40 OR #41 OR #42 OR #43 OR #44 OR #45 OR #46 OR #47 OR #48 OR #49 OR #50 OR #51 OR #52 130003

#54 5 AND #31 AND #53 in Cochrane Reviews 350

**Web of Science search**

(from All Databases)

You searched for: TOPIC: (mHealth OR smartphone OR mobile health OR telemedicin* OR App OR mobile application OR internet OR text message OR SMS OR wireless technology OR web-based OR acceleromet* OR wearable) AND TOPIC: (exercise OR physical activity OR movement OR sport OR healthy lifestyle OR health promotion OR active living OR fitness OR diet OR sedentary OR sitting OR nutrition OR vegetable OR fruit OR meal OR snack* OR healthy eating) AND TOPIC: (social OR friend* OR family OR social network OR school OR peer group OR social feature OR social network OR ecological momentary assessment OR just-in-time OR EMA OR EMI OR ecological momentary intervention OR trigger OR BCT OR behavio* change OR behavio* OR behavio* modification)

Refined by: DOCUMENT TYPES: ( REVIEW )

Timespan: 1990-2019. Databases: WOS, BIOSIS, KJD, MEDLINE, RSCI, SCIELO.

Search language=Auto
